# Recurrence of Dysplastic Nevi Is Strongly Associated with Extension of the Lesions to the Lateral Margins and into the Deep Margins through the Hair Follicles in the Original Shave Removal Specimens

**DOI:** 10.1155/2016/8523947

**Published:** 2016-09-28

**Authors:** Amin Maghari

**Affiliations:** DermOne Dermatology Associates of New Jersey, P.C. 540 Lacey Road, Forked River, NJ 08731, USA

## Abstract

Melanocytic nevi, including dysplastic or atypical nevi (DN), can recur or persist following shave removal procedures, and recurrence may resemble melanoma, both clinically and histologically (pseudomelanoma). Recurrence may originate from proliferation of the remaining neoplastic melanocytes following incomplete removal. The present study determines the rate and etiology of this event. A cross-sectional analysis of 110 excision specimens showing histological recurrence was performed, and these specimens were compared to the slides of the original shave specimens showing mildly atypical DN. In the second portion of the study, a retrospective review of 167 cases with biopsy-proven mildly atypical DN which were followed up for at least two years was conducted to determine the rate of recurrence/persistence. When followed up for two years, DN, with positive shave margins, defined by extension or very close extension (≤0.2 mm) of the lesions to the lateral margins and into the deep margins through the hair follicles in the shave removal specimens, have a higher probability of recurrence than DN with negative (or clear) margins (odds ratio (OR) = 158; 95% confidence interval (CI) = 36.62–683; *P* < 0.001). The overall rate of histologically confirmed recurrence/persistence was approximately 10%.

## 1. Introduction

Dysplastic or atypical nevi (DN) are one of the most frequently encountered lesions in dermatopathology. The prevalence of histological DN varies by race and ethnicity and can reach up to 50% in some white populations [1]. Not only are persons with DN at an increased risk of developing melanoma [2] but also DN can also serve as precursors for melanoma, particularly in the superficial spreading type [3], constituting the majority of melanomas [4]. DN are typically graded into three categories of mild, moderate, and severely atypical DN, which involve architectural atypia and cytological atypia. Architectural atypia includes lentiginous proliferation at the dermoepidermal junction extending beyond the dermal component (shoulder phenomena), elongation and bridging of adjacent epidermal rete ridges, and lamellar fibrosis in the papillary dermis, which is often accompanied by perivascular lymphocytic infiltrate. Cytological atypia is based on nuclear enlargement, hyperchromasia, uneven distribution of chromatin, presence of conspicuous cytoplasm with dusty pigmentation, and prominence of the nucleoli [5, 6].

Melanocytic nevi (including DN) can recur or persist following shave procedures, and recurrence may resemble melanoma, both clinically and histologically (pseudomelanoma). The majority of recurrences occur within less than six months of the primary shave procedure and only rarely occur after 24 months [7, 8]. This event may be due to proliferation of the remaining neoplastic melanocytes [9].

Until April 2015, in DermOne clinics (the location where this study was performed in Forked River, New Jersey), DN with moderate atypia or severe atypia on the shave removal specimens (SRS) were routinely excised regardless of the status of the margins, without providing sufficient time for recurrence. Patients with mildly atypical DN were followed up for up to two years, and the lesions were excised only if repigmentation occurred within or around the scars. However, since April 2015, DN with moderate atypia are excised only if the margins of the shave specimens are positive, and those with clear margins are only excised if they recur. The management guidelines for DN with mild atypia and severe atypia have remained the same.

Goodson et al. found a low (3-4%) rate of clinical recurrence for DN with mild atypia or moderate atypia and benign nevi when following up patients for two years after biopsy [10]. Excising mildly to moderately atypical DN usually does not result in a clinically significant change in diagnosis of the previous SRS, and the risk of transformation into melanoma is very low. DN with severe atypia, however, are more commonly associated with melanoma, and routine excision of biopsy-proven DN with severe atypia is beneficial for both detection of early melanomas and preventing them [11].

Although providers frequently ask pathologists to evaluate the margins in melanocytic neoplasms, a survey of more than 150 dermatopathologists revealed that only about one-third routinely comment on the margins of shave or punch specimens. This is probably due to the lack of guidelines for reporting margins of melanocytic neoplasms other than melanomas. Most pathologists, however, comment on the margins of the excision specimens (ES) [12].

In this study, the author compared the ES that showed evidence of recurrent or persistent DN (which were performed for repigmenting mildly atypical DN diagnosed on the SRS) with the slides of the original shave specimens to determine whether involvement of the margins is associated with recurrence.

## 2. Materials and Methods

A cross-sectional analysis of 110 ES of biopsy-proven mildly atypical DN from 87 patients in more than 20 clinical offices throughout the state of New Jersey between September 2014 and December 2014 was performed. The selected ES were elliptical or punch, showing histological evidence for recurrence (with or without residual lesions), and contained the scars of the previous shave procedures. The excision slides were recruited along with the slides of the original SRS. The selected shaves were accompanied by Melan-A immunohistochemical stain (IHCS) to highlight the subtle tumor cells that might otherwise have not been identifiable by the routine Hematoxylin and Eosin (H&E) stain. The SRS in which lateral or deep margins could not be assessed due to orientation artifacts such as twisted specimens, absence of full face, or missing lateral edges, as well as the cases without Melan-A IHCS, were not considered.

All tissues were fixed in formaldehyde (10%) for 12–36 hours and were embedded in paraffin, and Melan-A IHCS (clone A103, 1 : 25, Dako, Carpinteria, CA, USA) was applied on every case after antigen retrieval. UltraView universal detection kit with multimer technology method was used on an autostainer (Ventana Benchmark XT, Tucson, AZ, USA). Then, the slides were retrospectively reviewed by one dermatopathologist (the author) to identify the recurrent and residual lesions on the ES and to determine the involvement of the margins of the original SRS. The measurements were all performed using a calibrated eyepiece containing a graduated horizontal scale.

The case group was defined as 110 ES that showed recurrence (with or without residual lesions), and the control group was defined as the patients who did not develop clinical recurrence after two years of follow-up (110 mildly atypical DN diagnosed on 92 patients). Grading of DN was based on architectural atypia and cytological atypia. Cytological atypia for mildly atypical DN was based on nuclear size (about the size of a keratinocyte nucleus), hyperchromasia, and the lack of conspicuous nucleoli, abundant cytoplasm, or pagetoid spread, which are the histological features of higher grade DN and melanomas [5, 6]. Characteristic features of architectural atypia were discussed in Introduction.

In the ES (the case group), recurrence was defined as the presence of junctional melanocytic proliferation with or without upward migration, all limited within the boundaries of the underlying dermal scar, or the presence of dermal neoplastic melanocytes within the scar [13].

The presence of the neoplastic melanocytes in the adjacent unaltered epidermis or dermis was considered residual lesions. Dermal components showing benign nevus cells with histological maturation with descent located underneath the dermal scar were not considered as residual DN on ES but rather were considered as part of the background compound nevus.

On the original SRS, the true lateral borders of the lesions were defined as the last junctional melanocytic nest or as the last atypical melanocyte, either on H&E or on Melan-A IHCS. The true deep borders of the lesions were defined as the deepest extensions into the hair follicles or as the deepest dermal atypical or pigmented melanocytes (if there were any). Dermal histologically maturing melanocytes were not considered as the true deep borders of the lesions. The true lateral margins were defined as the edges of the specimens where both dermis and epidermis (dermoepidermal junction) were present (i.e., extension of the epidermis beyond the dermis was not considered as the true lateral margin).

**Figure 1:**
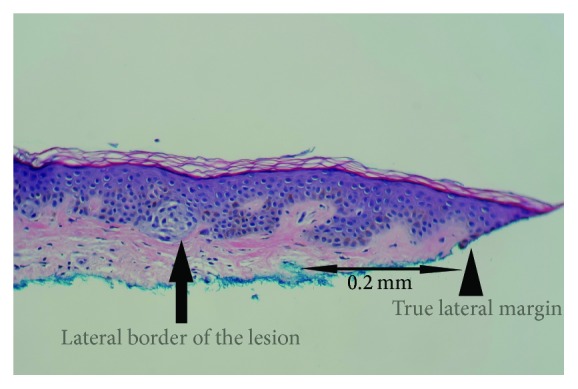
Clear Margin. True lateral border of the lesion: the last junctional melanocytic nest or the last atypical melanocyte (arrow). True lateral margin: the edge of the specimen where dermoepidermal junction is present (arrowhead) (H&E; 20x).

**Figure 2 fig2:**
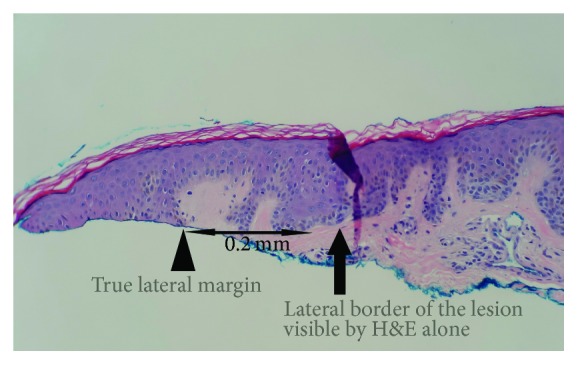
Margin appears clear (negative) on H&E stain (H&E; 20x).

**Figure 3 fig3:**
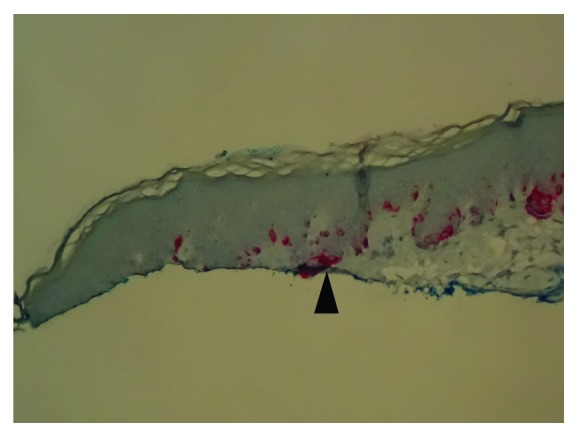
Melan-A highlighting subtle atypical melanocytes extending to the margin, not identifiable by the H&E stain in Figure 2 (arrowhead) (Melan A; 20x).

**Figure 4 fig4:**
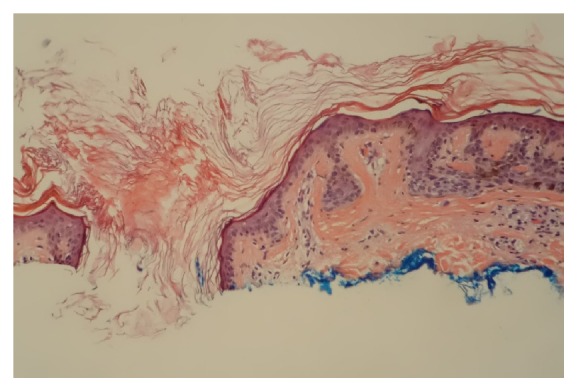
DN appears not to extend to the hair follicle (H&E; 20x).

**Figure 5:**
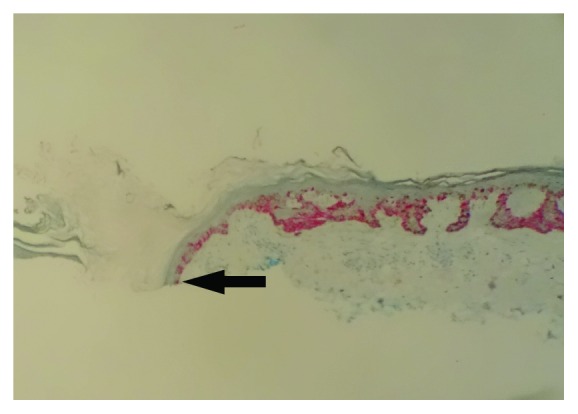
Melan-A highlighting atypical melanocytes extending to the hair follicle at the deep margin, not identifiable by the H&E stain in Figure 4 (arrow) (Melan A; 10x).

On the SRS, negative (or clear) margins were defined as neoplastic melanocytes in the junction, as well as the cells that extended to the hair follicles or the deepest dermal atypical or pigmented melanocytes, confined within >0.2 mm of the lateral and deep specimen margins. Melan-A IHCS was performed on all cases, which highlighted the subtle junctional tumor cells that may otherwise have not been identifiable by the routine H&E stains. Positive margins were defined as extension or very close extension (≤0.2 mm) of the lesional cells to the above-mentioned structures or if epidermal rete ridges containing nevus cells were transected superficially (see Figures 1 [Fig fig2] [Fig fig3] [Fig fig4]–5). A two by two contingency table was used to calculate the odds ratio.

In the second portion of this study, a retrospective review of 167 cases with biopsy-proven mildly atypical DN that were diagnosed between September and November 2013 and were followed up for two years was performed to determine the rate of recurrence.

This study was determined by Solutions IRB to be exempt from OHRP's Regulations for the Protection of Human Subjects (45 CFR 46) under category 4.

## 3. Results and Discussion

Evaluation of the original SRS of 110 ES showing recurrence (the case group) revealed that 108 cases (98.2%) had positive margins as follows: 25 cases had extension both into the hair follicles and into the lateral margins, 69 cases showed extension only to the lateral margins, 9 cases showed extension only to the hair follicles, and 5 cases had none of the above features; however, the epidermal rete ridges containing nevus cells were transected superficially. No shave specimen showed dermal atypical (or pigmented) melanocytes extending to the deep margins. Twenty-seven of the ES showed residual atypical nevi in addition to recurrent lesions.

Evaluation of the 110 SRS from patients who did not develop clinical recurrence after two years of follow-up (the control group) showed that 28 cases (25%) had positive margins as follows: twenty-three cases showed extension only to the lateral margins, 3 cases had extension both into hair follicles and into the lateral margins, and 2 cases showed extension only into the hair follicles at the deep margin. No cases with superficially transected epidermal rete ridges containing nevus cells were identified, and no dermal atypical (or pigmented) melanocytes extending to the deep margins were noted (see Table 1).

Of the 220 total shave specimens studied, in 68 cases (31%), Melan-A IHCS helped identify the true borders of the lesions which were not identifiable by the H&E stain alone.

Comparison between the original SRS and ES (see Table 2) showed a strong correlation between the junctional DN on SRS and junctional residual/recurrent lesion on the ES (97.4%, *P* < 0.001). The vast majority of the dermal components in the ES were located underneath the dermal scar (38 out of 40, 95%), showing histological maturation and representing leftover from the background compound nevus following the shave procedure rather than taking part in recurrence. However, they all showed junctional residual/recurrent lesion accounting for the repigmentation. In 2 ES, however, they were located within the scar, raising the possibility of occasional proliferation within the dermis. On the SRS of the case group (recurring DN), 17 cases showed maturing dermal nevus cells extending to the deep dermal (not follicular) margin. However, it is unlikely that they played a significant role in recurrence, since, in the control (nonrecurring) group, this did not result in recurrence (11 cases). Moreover, neither of the 2 cases that recurred despite having clear margins on SRS (as defined above) showed any maturing melanocytes extending to the deep dermal margins.

*Rate of Recurrence.* In the second portion of the study, a retrospective review of 167 cases with biopsy-proven mildly atypical DN was performed to determine the rate of recurrence regardless of the status of the original SRS margins. Patients were followed up for at least two years, and the study revealed the following: 23 cases (14%) demonstrated clinical recurrence/persistence when the sites of shaved DN were assessed and showed repigmentation. However, histological evaluation of H&E stained slides only confirmed the presence of recurrence/persistence in 17 cases (10.1%). The 6 cases where the author failed to detect histological evidence for recurrence/persistence showed either dermal hemorrhage (4 cases) or epidermal hyperpigmentation overlying the scar (2 cases). The interval between the shave procedure and recurrence ranged from 1 to 15 months, with the average time to recur of 5.5 months.

## 4. Conclusion

DN with positive margins defined by extension (≤0.2 mm) of the lesions to the lateral margins or into the deep margins through the hair follicles or when the epidermal rete ridges containing the lesional cells are transected superficially on the SRS have a statistically significant higher probability of recurrence than DN with negative (or clear) margins when followed up for up to 2 years (odds ratio (OR) = 158; 95% confidence interval (CI) = 36.62–683; *P* < 0.001). There was no statistically significant association between extension of the morphologically mature dermal melanocytes into the deep dermal margin and recurrence.

Follicular structures were present in only 81 shave specimens (37%), and 21 (26%) of them showed extension of the lesional cells into the deep margins. In the majority of those cases (15 cases, 71%), the neoplastic melanocytes were detected only by Melan-A IHCS. The author found Melan-A IHCS to be a useful marker to highlight the subtle neoplastic melanocytes at the dermoepidermal junction and their extension into the follicular structures, helping in identifying the true borders of the lesions that were not recognizable with the H&E stain.

As nevi (including dysplastic ones) mature in the dermis, they undergo senescence, defined by arrest in the proliferative capacity which is controlled by several mechanisms, such as telomere shortening, which is probably irreversible, and p16 expression. Senescence is accompanied by a number of morphological and functional changes, resulting in irreversible arrest in proliferation and pigment production of the nevus cells [14].

The data in this study differ from those of Sommer et al. [7] who found a higher percentage of recurrence due to deep margin involvement. This difference may be due to the fact that, in this study, a significant portion of the neoplastic melanocytes (31%) extending to the lateral margins were only identifiable by a melanocytic specific marker (Melan-A IHCS, which was not utilized in their study) and could be missed on H&E stain alone, resulting in a more significant association between the lateral margin involvement and recurrence. Otherwise, it is unlikely that morphologically mature and biologically permanently senescent melanocytes would play a significant role in recurrence. However, extension into the deep margins through the hair follicles was associated with recurrence in both studies.

Further, perhaps due to the higher numbers of recurrences and due to the utility of Melan-A IHCS, which detected more positive margins, the author found a more significant association between the margin involvement and recurrence compared to that found by Goodson et al. [10] who found only the method (shave technique in their study) and not the positive margins to be significantly associated with recurrence. Also, the overall rate of histologically confirmed recurrence/persistence in the current study was 10.1%, which is higher than that of Goodson et al. (3-4%) [10].

## Figures and Tables

**Table 1:** Excision specimens with recurrence compared with the original shave removal specimens.

	Case group (recurred) 110 cases	Control group (no recurrence or persistence) 110 cases
*Positive shave margins*	*108 (98.2%)*	*28 (25%)*
Lateral margins	69	23
Hair follicles	9	2
Lateral margins & hair follicles	25	3
Epidermal rete ridges transected superficially	5	0
Deep margins (pigmented or atypical cells)	0	0
*Negative (clear) margins*	*2 (1.8%)*	*82 (75%)*
* Deep margins (maturing cells, not considered true positive)*	*17*	*11*

**Table 2:** Comparison between the features of the original SRS and ES.

Original SRS	Junctional ES	ES compound
Junctional, 39	38	1
Compound, 71	40	31

## Additional Points

*Limitations.* This was a retrospective study, and since Melan-A IHCS was not performed on the ES, some of the subtle neoplastic cells may not have been identified by the H&E stains, thereby affecting the statistical values.

## References

[1] Crutcher W. A., Sagebiel R. W. (1984). Prevalence of dysplastic naevi in a community practice. *The Lancet*.

[2] Goldstein A. M., Tucker M. A. (2013). Dysplastic nevi and melanoma. *Cancer Epidemiology, Biomarkers & Prevention*.

[3] Bevona C., Goggins W., Quinn T., Fullerton J., Tsao H., Corona R. (2003). Cutaneous melanomas associated with nevi. *Archives of Dermatology*.

[4] Clark W. H., Elder D. E., Van Horn M. (1986). The biologic forms of malignant melanoma. *Human Pathology*.

[5] Arumi-Uria M., McNutt N. S., Finnerty B. (2003). Grading of atypia in nevi: correlation with melanoma risk. *Modern Pathology*.

[6] Culpepper K. S., Granter S. R., McKee P. H. (2004). My approach to atypical melanocytic lesions. *Journal of Clinical Pathology*.

[7] Sommer L. L., Barcia S. M., Clarke L. E., Helm K. F. (2011). Persistent melanocytic nevi: a review and analysis of 205 cases. *Journal of Cutaneous Pathology*.

[8] Fox J. C., Reed J. A., Shea C. R. (2011). The recurrent nevus phenomenon: a history of challenge, controversy, and discovery. *Archives of Pathology and Laboratory Medicine*.

[9] Sexton M., Sexton C. W. (1991). Recurrent pigmented melanocytic nevus: a benign lesion, not to be mistaken for malignant melanoma. *Archives of Pathology and Laboratory Medicine*.

[10] Goodson A. G., Florell S. R., Boucher K. M., Grossman D. (2010). Low rates of clinical recurrence after biopsy of benign to moderately dysplastic melanocytic nevi. *Journal of the American Academy of Dermatology*.

[11] Reddy K. K., Farber M. J., Bhawan J., Geronemus R. G., Rogers G. S. (2013). Atypical (dysplastic) nevi: outcomes of surgical excision and association with melanoma. *JAMA Dermatology*.

[12] Sellheyer K., Bergfeld W. F., Stewart E., Roberson G., Hammel J. (2005). Evaluation of surgical margins in melanocytic lesions: a survey among 152 dermatopathologist. *Journal of Cutaneous Pathology*.

[13] King R., Hayzen B. A., Page R. N., Googe P. B., Zeagler D., Mihm M. C. (2009). Recurrent nevus phenomenon: a clinicopathologic study of 357 cases and histologic comparison with melanoma with regression. *Modern Pathology*.

[14] Ross A. L., Sanchez M. I., Grichnik J. M. (2011). Nevus senescence. *ISRN Dermatology*.

